# Concurrent Papillary Thyroid Carcinoma and Anaplastic Thyroid Carcinoma Presenting With Hoarseness and Dysphagia

**DOI:** 10.7759/cureus.106319

**Published:** 2026-04-02

**Authors:** Sumreen Nazly, Arshia Ahmed, Salman J Khan, Gurdeep Singh

**Affiliations:** 1 Internal Medicine, University Medical and Dental College, Faisalabad, PAK; 2 Internal Medicine, Guthrie Lourdes Hospital, Binghamton, USA; 3 Public Health, University of Massachusetts Amherst, Amherst, USA; 4 Endocrinology, Diabetes and Metabolism, Our Lady of Lourdes Memorial Hospital, Binghamton, USA

**Keywords:** aggressive thyroid malignancy, anaplastic thyroid carcinoma, dedifferentiation, high grade thyroid cancer, mixed thyroid carcinoma, papillary thyroid carcinoma, poorly differentiated carcinoma, synchronous thyroid tumors, thyroid cancer transformation, thyroid tumor progression

## Abstract

Anaplastic thyroid carcinoma is a rapidly progressive thyroid malignancy associated with extensive local invasion and limited survival. We describe the case of a 60-year-old man who developed new-onset dysphagia and voice changes in the setting of a thyroid mass. Initial cytology and core sampling were consistent with papillary thyroid carcinoma, yet the patient’s accelerating compressive symptoms led to expedited total thyroidectomy. Surgical pathology ultimately demonstrated conventional papillary carcinoma with discrete foci of undifferentiated carcinoma within the same specimen.

This case highlights that rapidly progressive compressive symptoms in a patient with cytology-proven conventional papillary thyroid carcinoma may indicate an occult, unsampled anaplastic component, even in the absence of high-risk features on preoperative evaluation. The presence of both differentiated and undifferentiated components broadens the differential considerations regarding tumor evolution and highlights the need for close clinicopathologic correlation. Prompt recognition of alarming symptoms and early multidisciplinary coordination were central to management, particularly to mitigate the risk of airway compromise and to guide timely postoperative systemic therapy.

## Introduction

Anaplastic thyroid carcinoma (ATC) represents one of the most aggressive and lethal forms of thyroid malignancy, accounting for approximately 1-2% of all thyroid cancers but responsible for a disproportionate number of thyroid cancer-related deaths [1,2]. Despite advances in diagnostic and therapeutic modalities, the median survival remains approximately 3-6 months following diagnosis [1,3]. 

ATC may develop de novo or through dedifferentiation of a pre-existing well-differentiated thyroid carcinoma, most commonly papillary thyroid carcinoma (PTC), which typically follows an indolent clinical course with excellent long-term survival [4]. This transformation often manifests as a rapid increase in tumor size, compressive symptoms such as dysphagia, dyspnea, or hoarseness, and systemic manifestations like weight loss and malaise. In our case, the acute onset of compressive symptoms contrasted with the typically indolent course of PTC and ultimately reflected an underlying anaplastic component identified on final pathology. Recognition of this transformation is critical, as it dramatically alters both the prognosis and the therapeutic approach. 

Histologically, coexisting well-differentiated carcinoma is identified in a substantial proportion of ATC cases when surgical specimens are extensively sampled, most often of papillary type [5,6]. These tumors show a dual pattern, with typical papillary areas alongside undifferentiated pleomorphic or spindle cells. Molecular studies demonstrating shared mutations such as BRAF V600E, TERT promoter, and TP53 further confirm a clonal relationship and suggest a stepwise progression from differentiated to anaplastic carcinoma [7,8].

We present the case of a 60-year-old man with a two-month history of dysphonia and a one-month history of dysphagia, accompanied by unintentional weight loss of 15-20 pounds. Fine-needle aspiration showed PTC, and urgent total thyroidectomy was performed due to severe compressive symptoms, which revealed both PTC and ATC components.

## Case presentation

A 60-year-old man presented to the otolaryngology clinic with a two-month history of progressive hoarseness, accompanied by dysphagia to solids and liquids. He also reported significant unintentional weight loss of approximately 15-20 pounds over the preceding month. His symptoms had progressively worsened, prompting further evaluation. The patient had no significant past medical history and denied any prior surgeries or exposure to neck irradiation. He was a lifelong non-smoker. His family history was notable for multiple malignancies, including breast cancer in his mother, thyroid cancer in multiple female relatives on the maternal side, and pancreatic cancer in a paternal cousin.

On physical examination, a prominent, soft, right-sided neck mass measuring approximately 4-5 cm was palpated laterally to the thyroid gland. Initial laboratory evaluation, including complete blood count and basic metabolic panel, was unremarkable (Table 1). Thyroid functions were also unremarkable (Table 2). 

**Table 1:** Initial laboratory evaluation showing no significant abnormalities on complete blood count or basic metabolic panel

Parameter	Result	Reference Range
White blood cell count (WBC) (k/µL)	6.44	4.23-9.07
Red blood cell count (RBC) (M/µL)	5.03	4.30-5.89
Hemoglobin (g/dL)	14.0	13.7-17.5
Hematocrit (%)	42.2	40.1-51.0
Mean corpuscular volume (MCV) (fL)	83.9	79.0-92.2
Mean corpuscular hemoglobin (MCH) (pg)	27.8	25.7-32.2
Mean corpuscular hemoglobin concentration (MCHC) (g/dL)	33.2	32.3-36.5
Platelets (k/µL)	186	163-337
Mean platelet volume (MPV) (fL)	10.2	9.0-12.4
Red cell distribution width (RDW) (%)	13.5	11.6-14.4
Nucleated RBC (nRBC) (%)	0.0	0.0-0.2
Sodium (mmol/L)	138	136-145
Potassium (mmol/L)	3.9	3.5-5.1
Chloride (mmol/L)	103	98-110
Bicarbonate (CO₂) (mmol/L)	27	22-29
Calcium (mg/dL)	8.9	8.4-10.4
Albumin (g/dL)	3.8	3.5-5.2
Blood urea nitrogen (BUN) (mg/dL)	14	8-23
Creatinine (mg/dL)	0.79	0.67-1.17
Estimated GFR (eGFR) (mL/min/1.73 m²)	102	>60
BUN/Creatinine ratio	18	6-22
Glucose (mg/dL)	112	70-99
Total protein (g/dL)	6.8	6.4-8.3
Total bilirubin (mg/dL)	1.80	0.00-1.10
Aspartate aminotransferase (AST) (U/L)	18	0-40
Alanine aminotransferase (ALT) (U/L)	21	5-41
Alkaline phosphatase (U/L)	80	40-129
Anion gap (mmol/L)	8	3-11
Albumin/Globulin (A/G) ratio	1.3	0.8-2.0

**Table 2:** Thyroid function and antibody panel demonstrating euthyroid status with negative thyroid autoantibodies

Test	Result	Reference Range
Total T4	8.4	Normal
Total T3	0.78	Normal
Thyroid-stimulating hormone (TSH) (mIU/L)	1.23	0.4-4.5
Thyroglobulin antibodies	23.8	0-115
Thyroid peroxidase antibodies	10.7	0-34

**Figure 1:**
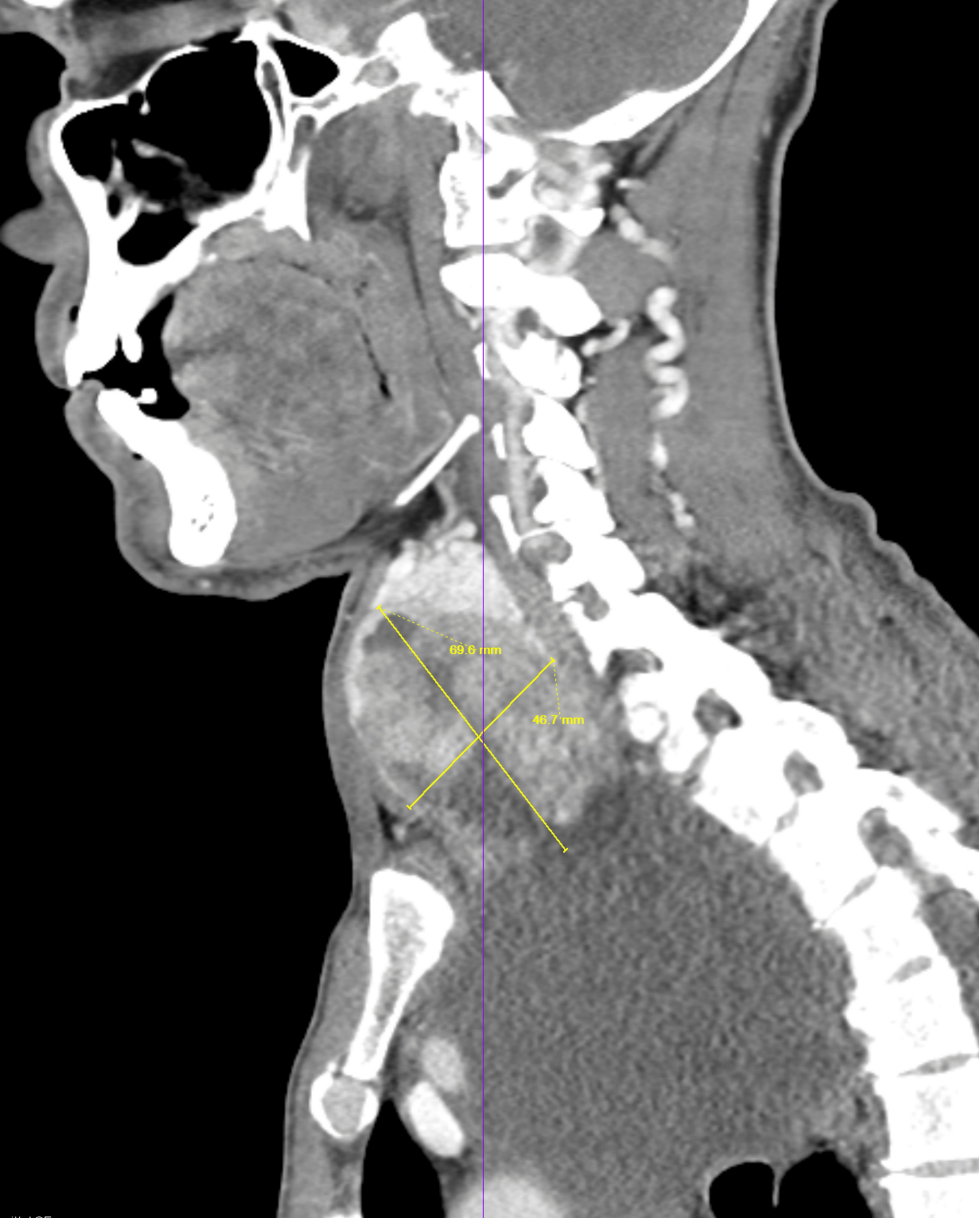
CT neck and chest showing a large right thyroid mass CT scan of the neck and chest demonstrating a large right thyroid mass measuring approximately 7.5 cm with features suspicious for malignancy. CT, computed tomography.

**Figure 2:**
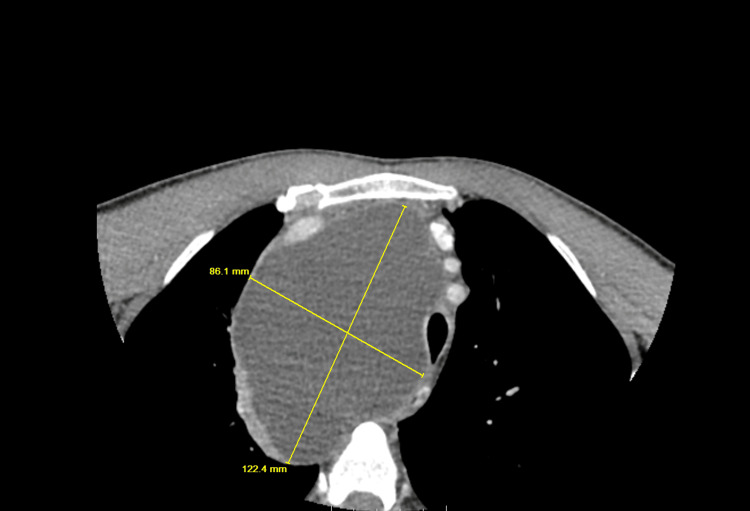
CT neck and chest showing a thyroid mass with mediastinal extension and mass effect CT of the neck and chest demonstrates a large cystic mediastinal extension measuring up to 12.2 cm, likely representing a cystic component of the thyroid mass, with a significant mass effect causing displacement of the larynx, trachea, and esophagus. CT, computed tomography.

**Figure 3:**
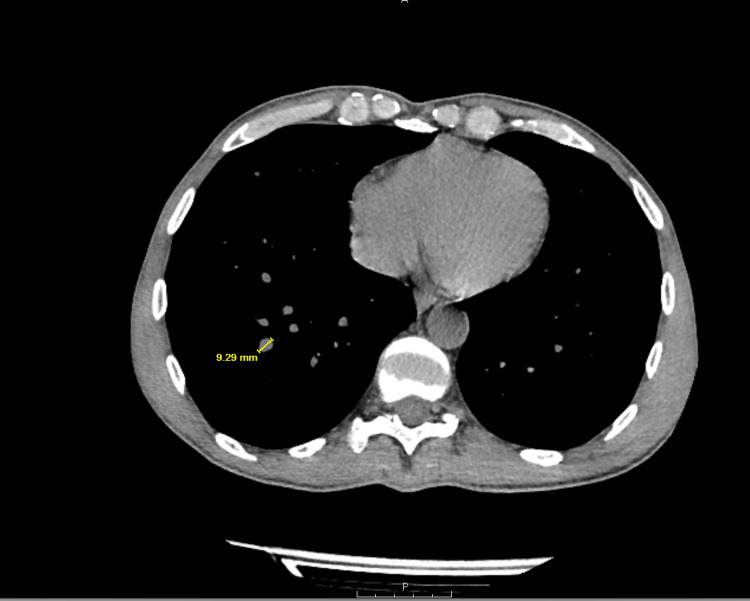
CT chest showing multiple bilateral pulmonary nodules CT of the chest demonstrates multiple bilateral pulmonary nodules concerning for metastatic disease. CT, computed tomography.

Given the presence of dysphonia and a palpable neck mass, the otolaryngology team recommended further diagnostic workup. Computed tomography (CT) of the neck and chest revealed a markedly enlarged right thyroid mass measuring approximately 7.5 cm with imaging features suspicious for malignancy (Figure 1). In addition, a large cystic extension into the mediastinum measuring up to 12.2 cm was identified, likely representing a cystic component of the same lesion, for which tissue sampling was recommended. The mass exerted a significant mass effect, resulting in compression and leftward deviation of the larynx, trachea, and esophagus (Figure 2). Multiple bilateral pulmonary nodules measuring up to 10 mm were also noted, raising concern for metastatic disease (Figure 3). 

Further diagnostic evaluation was pursued, given the alarming clinical and radiographic findings. Fine-needle aspiration of the thyroid lesion confirmed PTC, classified as Bethesda Category VI [9]. 

As his swallowing difficulty progressed, a formal swallow study was obtained, which demonstrated moderate oropharyngeal dysphagia with significant aspiration risk. Given the imminent threat to airway protection, the patient underwent surgical evaluation. Airway stabilization strategies, including possible endotracheal intubation and tracheostomy, were discussed with the patient; however, he preferred to defer invasive airway intervention unless absolutely necessary. In light of the tumor’s complexity and extent, transfer to an academic tertiary care center was recommended for specialized surgical and oncologic management.

During hospitalization, the patient’s severe dysphagia and ongoing weight loss necessitated placement of a percutaneous endoscopic gastrostomy (PEG) tube to ensure adequate nutritional support. A core biopsy of the right thyroid mass obtained during admission, along with immunohistochemical analysis, again confirmed PTC, reinforcing the diagnosis. Due to compression of the trachea, larynx, and esophagus, a large cystic extension into the mediastinum was successfully drained.

Subsequent positron emission tomography-computed tomography (PET-CT) imaging revealed metabolically active disease consistent with advanced thyroid malignancy, including mediastinal extension and multiple pulmonary metastases, highlighting the aggressive clinical behavior of the tumor. 

At follow-up with endocrinology, the patient was counseled extensively regarding the severity of his disease and the need for thyroidectomy. As part of the preoperative assessment, surgical oncology recommended an MRI of the neck, which reassuringly demonstrated no direct invasion of the larynx, trachea, or cervical esophagus.

With airway and nutritional status stabilized, the patient proceeded to a near-total thyroidectomy. His postoperative recovery was remarkable. Pain was well controlled, and his voice remained clear without hoarseness, alleviating earlier concerns regarding recurrent laryngeal nerve involvement. 

Final histopathologic examination revealed classic PTC with focal areas of anaplastic carcinoma, positive surgical margins, absence of vascular or lymphatic invasion, and a negative level VI lymph node, corresponding to pT3aN0 disease according to the AJCC 8th edition TNM staging system [10]. Clinically, the impact of surgery was profound. Within two weeks, the patient reported a dramatic improvement in appetite and swallowing, accompanied by a 10-pound weight gain. 

Despite these gains, biochemical follow-up one month after surgery revealed a markedly elevated thyroglobulin level of 230 ng/mL, raising concern for persistent or residual disease. Endocrinology recommended close follow-up with medical and radiation oncology. Given the presence of an anaplastic component, Hematology/Oncology advised initiation of systemic therapy. Comprehensive molecular testing, including next-generation sequencing and Guardant 360 analysis, was performed, which returned negative for NTRK, ALK, RET, and BRAF mutations. Based on these results, Lenvatinib was initiated as a vascular endothelial growth factor (VEGF)-targeted therapy, and the patient has tolerated therapy reasonably well without major adverse effects, aside from mild fatigue and arthralgias, which remain manageable. Follow-up CT of the neck, chest, abdomen, and pelvis demonstrated a decrease in the size and number of pulmonary metastases, indicating a favorable response to treatment.

## Discussion

The concurrent presence of PTC and ATC within the same thyroid gland represents a diagnostically and clinically significant entity [11]. Coexisting differentiated carcinoma is frequently identified in ATC surgical specimens, with studies reporting rates ranging from 30% to over 70%, depending on the extent of sampling [5,6]. This dual morphology provides morphologic evidence of potential stepwise dedifferentiation and carries important prognostic and therapeutic implications [11]. 

Our case adds to the existing literature by demonstrating that a focal (minor) anaplastic component may evade detection on preoperative cytology and core biopsy and instead present clinically through rapidly progressive compressive symptoms. The discordance between conventional PTC on biopsy and aggressive clinical behavior underscores the need to maintain suspicion for occult dedifferentiation when the clinical course is disproportionate to initial pathology findings.

In our patient, the initial fine-needle aspiration and subsequent core biopsy revealed PTC. However, the presence of aggressive compressive symptoms and a rapidly progressive clinical course raised suspicion that the diagnosis might not be limited to conventional papillary carcinoma. Postoperative histopathological examination revealed classic PTC with focal areas of ATC, highlighting dual histology within a single thyroid gland. In this case, it remains unclear whether the anaplastic component arose de novo or through dedifferentiation from pre-existing PTC, as preoperative sampling demonstrated only papillary histology and may have missed a focal anaplastic component. Therefore, we describe this as concurrent PTC and ATC.

The relationship between coexisting PTC and ATC components remains an area of active investigation. While molecular studies demonstrate shared mutations supporting a dedifferentiation model, the possibility of independent origins should also be considered [7,8]. The absence of a clearly identifiable transitional zone in some tumors, along with aggressive clinical presentations disproportionate to initial biopsy findings, underscores the biological heterogeneity of thyroid malignancies and the need to correlate clinical behavior with histopathology [1,12]. 

Molecular profiling suggests that progression from PTC to ATC involves sequential accumulation of genetic alterations [7,13]. While PTC is typically driven by MAPK pathway mutations, transformation to ATC involves additional alterations, including TERT promoter mutations, TP53 inactivation, and PI3K/AKT/mTOR pathway dysregulation [1,7]. TERT promoter mutations, in particular, appear to represent an independent risk factor for anaplastic transformation [8].

Management of ATC requires an urgent multidisciplinary approach involving endocrinology, oncology, surgery, radiation oncology, and palliative care [1]. For patients with disease confined to the thyroid without major structural invasion, complete surgical resection remains the cornerstone of therapy, typically followed by adjuvant external-beam radiation and systemic therapy [1]. 

A major therapeutic advance has been the identification of BRAF V600E mutations, present in approximately 25-50% of ATC cases [1,7]. The combination of dabrafenib and trametinib has demonstrated significant response rates, with an overall response of 56% and a median survival of 14.5 months in the ROAR study [14]. 

Beyond BRAF, genomic profiling has identified additional alterations such as RET and NTRK fusions, while PD-L1 overexpression provides a rationale for immunotherapy approaches [1]. These developments underscore the importance of early genomic testing to guide precision-based therapy. 

In our patient, next-generation sequencing and Guardant360 testing were negative for actionable mutations, leading to initiation of lenvatinib. Lenvatinib, a multikinase inhibitor targeting VEGFR, FGFR, PDGFR-α, RET, and KIT, has demonstrated activity in mutation-negative or unresectable ATC [15]. Its integration into multimodal therapy has shown encouraging outcomes in selected patients [16]. 

Survival among patients with coexisting PTC and ATC lies between that of pure PTC and pure ATC and depends largely on aggressive clinicopathologic features [9]. Emergence of rapid tumor progression, compressive symptoms, or loss of radioactive iodine responsiveness should prompt urgent reassessment and multidisciplinary evaluation [1]. 

Supportive and palliative care remain essential. Given the high risk of airway compromise, early airway planning, nutritional support, pain control, and psychosocial care are critical components of management [1].

## Conclusions

This case highlights the diagnostic and therapeutic challenges of ATC, particularly when coexisting with PTC. The sudden onset of red-flag symptoms, such as a rapidly enlarging neck mass, dysphagia, hoarseness, or unintentional weight loss, should prompt urgent evaluation and multidisciplinary intervention. Temporizing measures, including percutaneous drainage of cystic components, may help stabilize the patient and facilitate definitive surgical management. Despite successful resection and functional recovery, the presence of an anaplastic component necessitates early initiation of systemic therapy and close postoperative surveillance. Overall, this case emphasizes the importance of early recognition, individualized stepwise management, and a coordinated multidisciplinary approach to optimize outcomes in patients with aggressive thyroid malignancies.

## References

[1] Bible KC, Kebebew E, Brierley J (2021). 2021 American Thyroid Association guidelines for management of patients with anaplastic thyroid cancer. Thyroid.

[2] Boucai L, Zafereo M, Cabanillas ME (2024). Thyroid cancer: A review. JAMA.

[3] Fagin JA, Wells SA Jr (2016). Biologic and clinical perspectives on thyroid cancer. N Engl J Med.

[4] Haugen BR, Alexander EK, Bible KC (2016). 2015 American Thyroid Association management guidelines for adult patients with thyroid nodules and differentiated thyroid cancer: The American Thyroid Association Guidelines Task Force on thyroid nodules and differentiated thyroid cancer. Thyroid.

[5] Maniakas A, Dadu R, Busaidy NL (2020). Evaluation of overall survival in patients with anaplastic thyroid carcinoma, 2000-2019. JAMA Oncol.

[6] Molinaro E, Romei C, Biagini A (2017). Anaplastic thyroid carcinoma: From clinicopathology to genetics and advanced therapies. Nat Rev Endocrinol.

[7] Chen DW, Lang BHH, McLeod DSA, Newbold K, Haymart MR (2023). Thyroid cancer. Lancet.

[8] Oishi N, Kondo T, Ebina A (2017). Molecular alterations of coexisting thyroid papillary carcinoma and anaplastic carcinoma: Identification of TERT mutation as an independent risk factor for transformation. Mod Pathol.

[9] Cibas ES, Ali SZ (2017). The 2017 Bethesda System for reporting thyroid cytopathology. Thyroid.

[10] Amin MB, Edge SB, Greene FL (2017). AJCC Cancer Staging Manual. https://link.springer.com/book/10.1007/978-3-319-40618-3.

[11] Greenberg JA, Moore MD, Thiesmeyer JW (2023). Coexisting papillary and anaplastic thyroid cancer: Elucidating the spectrum of aggressive behavior. Ann Surg Oncol.

[12] Capdevila J, Mayor R, Mancuso FM (2018). Early evolutionary divergence between papillary and anaplastic thyroid cancers. Ann Oncol.

[13] Landa I, Ibrahimpasic T, Boucai L (2016). Genomic and transcriptomic hallmarks of poorly differentiated and anaplastic thyroid cancers. J Clin Invest.

[14] Subbiah V, Kreitman RJ, Wainberg ZA (2022). Dabrafenib plus trametinib in patients with BRAF V600E-mutant anaplastic thyroid cancer: Updated analysis from the phase II ROAR basket study. Ann Oncol.

[15] Higashiyama T, Sugino K, Hara H (2022). Phase II study of the efficacy and safety of lenvatinib for anaplastic thyroid cancer (HOPE). Eur J Cancer.

[16] Dierks C, Seufert J, Aumann K (2021). Combination of lenvatinib and pembrolizumab is an effective treatment option for anaplastic and poorly differentiated thyroid carcinoma. Thyroid.

